# Effectiveness of implementing family involvement on patient outcomes in individuals with psychotic disorders: a pragmatic cluster randomised controlled trial

**DOI:** 10.1186/s12888-025-07501-z

**Published:** 2025-12-04

**Authors:** Irene Norheim, Reidar Pedersen, Maria Lie Selle, Jan Ivar Røssberg, Lars Hestmark, Kristin Sverdvik Heiervang, Torleif Ruud, Vilde Maria Åsholt, Kristiane Myckland Hansson, Paul Møller, Roar Fosse, Solveig Helene Høymork Kjus, Maria Romøren

**Affiliations:** 1 Department of Mental Health Research and Development, Division of Mental Health and Addiction, Vestre Viken Hospital Trust, Drammen, Norway; 2 Centre for Medical Ethics, Institute of Health and Society, University of Oslo, Oslo, Norway; 3 Health Services Research Unit, Akershus University Hospital, Lørenskog, Norway; 4 Section for Treatment Research, Division of Mental Health and Addiction, Oslo University Hospital, Oslo, Norway; 5 Institute of Clinical Medicine, University of Oslo, Oslo, Norway; 6 Division of Mental Health Services, Akershus University Hospital, Lørenskog, Norway

**Keywords:** Interpersonal relationships, Psychosis, Schizophrenia, Family intervention, Family psychoeducation, Evidence-based practice, Mental health services, Cluster randomised controlled trial

## Abstract

**Background:**

The purpose of this sub-study, as part of the *Implementation of guidelines on Family Involvement for persons with Psychotic disorders (IFIP)* trial, was to examine the effectiveness of family interventions (FIs) implemented through an implementation support program (ISP) on patients’ outcomes.

**Methods:**

The IFIP trial is a cluster randomised controlled trial where 15 Community Mental Health Centre units were randomly allocated either to the ISP, which included FIs as clinical interventions, implementation strategies, and implementation interventions, or to treatment as usual (TAU). In the control arm, patients could have access to FIs. Patients with psychotic disorders in both arms were asked to complete questionnaires at inclusion, 6- and 12 months. The primary outcome was patients’ difficulties in interpersonal relationships measured with the Relationships subscale of Behaviour and Symptom Identification scale. The secondary outcomes were patients’ mental health and functioning, perceived expressed emotions, recovery, and quality of life. Linear mixed-effects models were used to analyse differences in change over time between the two arms.

**Results:**

We included 231 patients (135 from the intervention units; 96 from the control units). Except for the first 24% of the 6 months questionnaires, the 6- and 12-month data were collected during the COVID-19 pandemic. There were no significant differences in the primary outcome, difficulties in interpersonal relationships at 12 months, between the intervention and control arms (Cohen’s d = -0.19, *p* = 0.2). The secondary outcomes showed less perceived criticism from the relatives (Perceived Criticism sub-score; d = − 0.4, *p* = 0.01) and reduced difficulties in overall functioning (Health of the Nation Outcome Scale; d = − 0.29, *p* = 0.01) in the intervention arm compared to the control arm at 12 months.

**Conclusion:**

The FIs, implemented through the ISP, did not achieve significant decreases in patients’ difficulties with interpersonal relationships. However, this sub-study indicates that FIs in routine mental health practices can nurture a more positive family environment and enhance the patient’s process toward improved functioning. The complexity of the study design, and the restrictions due to COVID-19, may have diminished the implementation and effectiveness of the measured FIs for the patients.

**Trial registration:**

ClinicalTrials.gov Identifier NCT03869177. Registered 2019-02-28.

**Supplementary Information:**

The online version contains supplementary material available at https://doi.org/10.1186/s12888-025-07501-z.

## Introduction

Psychotic disorders, often emerging early in life, can lead to long-term social, cognitive, and occupational impairments [1]. Psychosis and its symptoms can impact the individual’s everyday life, causing difficulties in social relationships, communication, and self-expression, and leading to stigma [2]. Schizophrenia ranks among the top 15 leading causes of disability worldwide [1, 3], making it crucial to highlight the best possible diagnostics, treatment, and follow-up [4, 5]. The individual’s family is often considerably affected by concerns, stress, and caregiver burden, impacting their daily lives, health, and quality of life [6–9]. Early detection combined with medical and psychosocial treatments improve prognosis and support recovery and remission [10–14].

Family interventions (FIs) can lead to reductions in symptoms and inpatient stays [15–18], relapse rates [19–22], and improve treatment satisfaction and compliance [16]. Additionally, FIs may enhance social, clinical and overall functioning [16, 17, 19, 20, 22, 23], quality of life [16, 24], and employment outcomes [25–27], while also lowering levels of expressed emotions among family members [28–30].

FIs are complex interventions, and we still do not know enough about the mechanisms that lead to changes. Specifically, a study by Kuipers et al. emphasized the importance of FIs providing opportunities for emotional changes. The key elements of FIs, which involve replacing stress, anxiety, and criticism with a greater level of tolerance and increased problem-solving and coping strategies involving the patient, are assumed to lead to an improved emotional climate [31, 32]. In particular, family psychoeducation (FPE) has been found to improve social relations [23], family relationships [33], and reduce friction in interpersonal relationships [34–36] among patients with schizophrenia. Enhancement of the family environment is considered a mediator for patient outcomes [30]. It appears to occur through FIs, which contribute to increased family competence (such as changes in knowledge, attitudes, and skills) and facilitate the family’s recovery by reducing family burden and promoting mental health [16].

Structured FIs, like FPE, are a key component in evidence-based treatment for individuals with psychoticdisorders [12, 20, 37, 38] and are endorsed in treatment guidelines internationally [39–42]. In Norway, the national guide for relatives within health and care services [43] recommends basic family involvement and support practices (BFIS) for all groups of relatives, regardless of the patient’s or service user’s diagnosis. This guidance considers family members of varying ages – including the elderly, adults, adolescents, and children - as relatives. National treatment guidelines for individuals with psychotic disorders recommend FPE as a first-line treatment [44] (currently under review).

Despite these endorsements, research consistently shows that the implementation rate of FIs remains poor within routine mental health care, leaving a large knowledge-practice gap [45–50]. Large-scale implementation studies of FIs to overcome barriers and support sustainability are absent [51]. When prior studies have examined FIs at the individual level, without systematic and overarching system implementation, the complexities of implementation and its sustainability have not been addressed, limiting the generalizability for patients and their families [52]. The GET UP PIANO and the NIMH RAISE ETP trials have investigated the effectiveness of a comprehensive team-based treatment approach, which included FPE, for first-episode psychosis patients. Both studies found improvements in patients’ clinical outcomes [53, 54], and the NIMH RAISE ETP trial also found improved quality of life and functioning [54].

The aim of the large-scale non-blinded pragmatic *Implementation of guidelines on Family Involvement for persons with Psychotic disorders (IFIP)* trial was to increase the implementation of the national guidelines in Community Mental Health Centres (CMHC) and thereby improve outcomes for patients and their family members [55]. The project group developed and utilized a comprehensive Implementation Support Program (ISP) alongside FIs (including BFIS and FPE). The interventions and their implementation were evaluated using a hybrid effectiveness-implementation cluster randomised controlled trial (c-RCT) design, combined with a mixed methods design. The project group has published several research articles related to the implementation and evaluation of FIs within the IFIP trial (Table 1).

**Table 1:** Research articles published in the IFIP trial

Type of study assessed	Methods	The titles with reference number
NA	Study protocol	Implementation of guidelines on family involvement for persons with psychotic disorders in community mental health centres (IFIP): protocol for a cluster randomised controlled trial (55)
Implementation	Fidelity measurements	Family involvement practices for persons with psychotic disorders in community mental health centres – a cross-sectional fidelity-based study (46)
Implementation	Fidelity measurements	Implementation of Guidelines on Family Involvement for Persons with Psychotic Disorders (IFIP): A Cluster Randomised Controlled Trial (57)
Implementation	Qualitative interviews and focus groups with clinicians and leaders	Barriers and facilitators when implementing family involvement for persons with psychotic disorders in community mental health centres–a nested qualitative study (47)
Implementation	Qualitative interviews and focus groups with clinicians and leaders	The duty of confidentiality during family involvement: ethical challenges and possible solutions in the treatment of persons with psychotic disorders (108)
Clinical	Qualitative interviews with clinicians	Clinicians’ perceptions of family involvement in the treatment of persons with psychotic disorders: a nested qualitative study (90)
Clinical	Qualitative interviews with patients	“The most important thing is that those closest to you, understand you”: a nested qualitative study of persons with psychotic disorders’ experiences with family involvement (89)
Clinical	Questionnaires to relatives	Implementation of guidelines on Family Involvement for persons with Psychotic disorders: a pragmatic cluster randomized trial. Effect on relatives’ outcomes and family interventions received (56)
Clinical	Questionnaires to patients and clinicians (the current article)	Effectiveness of implementing family involvement on patient outcomes in individuals with psychotic disorders: a pragmatic cluster randomised controlled trial.

The ISP, which guided the implementation of the national guidelines for family involvement, was the main difference between the intervention and control arms. The primary objective of the IFIP trial was to assess whether the ISP, implemented in the CMHC units (clusters) in the intervention arm, was associated with a higher level of adherence to the national guidelines compared to CMHC units in the control arm. A secondary objective was to evaluate the effectiveness of FIs, implemented through the ISP, on selected outcomes for included patients and their relatives in the intervention and control arm. The outcomes for relatives have been published previously [56].

Due to the study design and adherence to the intention-to-treat principle, all patients and their relatives recruited in both the intervention and control arms were included in the analyses of the effectiveness of the clinical interventions, regardless of the specific treatment received. It is noteworthy that the implementation of family involvement practices was not uniformly successful across all participating CMHC units within the intervention arm. This methodological approach provides an evaluation of the comprehensive impact of enhanced family involvement practices in routine clinical practice among all eligible patients at the CMHC units, rather than under more controlled conditions or confining analyses to those directly receiving FIs.

### Research questions

In this effectiveness study of FIs implemented through an ISP, we wanted to answer the following research question: Did patients in the intervention arm experience an improvement in outcomes compared to those in the control arm? The primary outcome was patients’ difficulties in interpersonal relationships. The secondary outcomes were patients’ mental health and functioning, perceived expressed emotions, recovery, and quality of life.

## Methods

### Setting and randomization at the cluster level

Five Health Trusts in South-Eastern Norway participated in the IFIP trial, comprising 15 CMHC units. The CMHC units served a catchment area of 1.3 million residents. Detailed information about the 15 participating CMHC units [46] and their division into 14 clusters has been published [57]. The research group initiated the Implementation Support Program (ISP) in February 2019 and concluded it in November 2021 across the seven clusters allocated to the intervention arm.

### Design for the patient outcome sub-study

The current sub-study reports the effectiveness of the clinical interventions (FIs), implemented through the ISP, for patients in the intervention arm relative to the patients receiving TAU. This article adheres to the “Consolidated Standards of Reporting Trials (CONSORT) statement 2010: extension to cluster randomised trials” [58] (Supplementary File 1).

The IFIP study protocol [55] and the relative outcomes article [56] offer detailed descriptions of the design and methods employed in the current sub-study. Consequently, only a brief overview will be presented.

### Recruitment procedures and participants at the individual level

The participating CMHC units in both intervention and control arms included patients with psychotic disorders, along with one close relative, for the two effectiveness studies [55]. Recruitment spanned from March 2019 to September 2020, after the completion of the initial ISP training in February 2019 [56]. Patients were invited to participate in the sub-study, providing written informed consent for their participation and allowing clinicians to contact a designated close relative [55].

The CMHC units each provided treatment to between 28 and 217 patients with a psychotic disorder, amounting to a total of 1392 patients [46]. We included patients aged 18 years or older; with a confirmed or provisional diagnosis of a psychotic disorder (ICD-10 codes F20-29), certain enough to begin treatment. Exclusion criteria encompassed being a forensic client; inability to provide consent; lack of relatives; or having previously completed extensive FPE sessions (more than five single-family or ten multi-family group sessions) [55].

Patient/relative pairs were recruited for both study arms regardless of the decision to offer FIs or other treatments, with no requirement for specific intervention or support during the 12 months following inclusion [55]. Likewise, involvement in FIs did not necessitate participation in the study. In addition to assess the impact of the intervention in a naturalistic setting, the strategy is ethically sound by not restricting the recommended treatment only to patients eligible and willing to participate in the study [55].

### Intervention programme

**Fig. 1:**
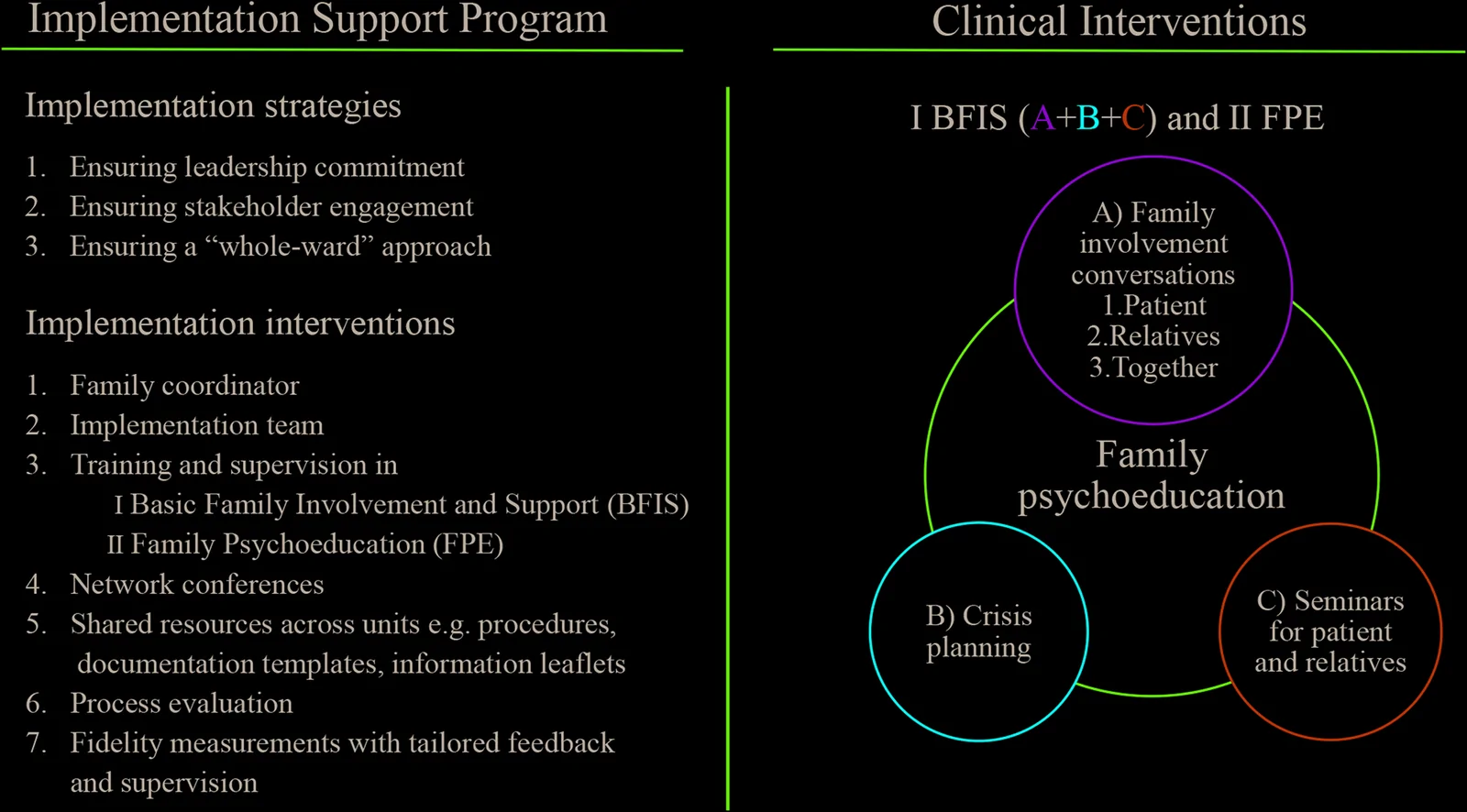
The implementation support program and the family interventions in the IFIP trial

The clinical interventions were implemented through the ISP (Fig. 1) on the level of CMHC units in the intervention arm as an add-on intervention to TAU. This approach was strategically employed to enhance both the quality and quantity of FIs. A detailed account of the ISP has been published elsewhere [57].

The clinical interventions, previously described in the relative outcomes article [56], were developed to include two tiers of family involvement, in accordance with the national guidelines [43, 44]: (1) Basic family involvement and support (BFIS) [57] to ensure conversations with both the patient and the relative(s) about the importance of involving the family in the treatment, offering FPE, providing psychoeducative seminars and preparing a crisis plan, (2) FPE based on McFarlane’s family work [59], customized for single-family groups with separate alliance sessions, followed by joint sessions with the patient and the relative(s) together. FPE encompasses emotional support, psychoeducation, crisis management, and communication and problem-solving skills. The duration of single-family groups generally spans 9 to 12 months, with adjustments made according to family needs. The strategy for the clinical interventions involved clinicians offering BFIS to all patients and their relatives and delivering FPE to as many as feasible [56, 57].

After the pandemic lockdown in Norway on the 12th of March 2020, the government implemented strict measures to limit the infection, e.g. closure of kindergartens and schools, access control in healthcare institutions, and encouragement to avoid public transport as much as possible [60]. The government encouraged health services to utilise digital platforms, including video consultation [61]. While some participating CMHC units successfully adapted to these changes, most encountered challenges due to a combination of restrictions, staff shortages, and difficulties with digital modalities. As a result, BFIS and FPE were either postponed, reduced, modified (e.g. such as shifting from in-person to digital meetings), concluded earlier than intended, or not commenced at all [56].

### Treatment as usual (TAU)

Patients in the control group received standard treatment from clinicians in the CMHC units. Treatment as usual encompassed a variety of therapeutic orientations employed within Norwegian specialised mental health services. We did not evaluate the content or quality of any structured treatment approaches. Consultations with the next of kin mainly occurred in a random manner, driven by the relatives’ requests, the clinicians’ interest, and their expertise, with or without the patient’s presence. The patients were rarely, if ever, offered FPE.

### Outcome measures

#### Baseline data collection

The patients’ sociodemographic information, self-reported adherence to prescribed medication, and level of functioning measured with the Global Assessment of Functioning scale, split version (GAF – Split version) [62, 63] were collected as background variables. The GAF Split version is a 2 items scale for symptoms (GAF-S) and functioning (GAF-F) with scores ranging from one to hundred (lower value, more severe symptoms/functional impairments). The validity of the separate GAF dimensions has been confirmed by discriminant and concurrent associations with other relevant clinical measures [64], and both symptom and function scores have proven to be highly consistent across experienced raters [65].

#### Service level outcomes

The effectiveness of FIs, implemented through the ISP, on patient outcomes must be understood in the context of FIs and crisis plans provided at the service level. Therefore, we briefly describe both previously published and new service level outcomes data in the current paper (results). The data collection of these outcomes are described in the relative outcomes article [56] and are summarised below.

Service level outcomes were assessed by the clinicians in terms of whether the patient had a crisis plan and had participated in FPE, including the number of FPE sessions, at baseline and 12 months. Clinicians had access to medical records, from which service level data for “the last 12 months” were obtained. Relatives reported participation in supportive or next of kin consultations within the CMHC units. At baseline, they detailed their involvement over “the last 12 months”, as well as any previous participation. At 6 months and 12 months, relatives reported “participation within the last 6 months”.

#### Clinical outcomes

The following patient outcome measures were assessed at baseline, 6 months, and 12 months, and by the patients’ clinicians at baseline and 12 months. Outcome of interest was defined as difference from baseline to 6 months and 12 months for the patient-reported questionnaires, and from baseline to 12 months for the clinician-reported questionnaires. The reasoning for selecting outcome measures, along with comprehensive details of all measures, is detailed in the IFIP study protocol [55].

#### The primary outcome for patients

##### The Relationships subscale from the Behavior and symptom identification scale (BASIS-24)

The Relationships subscale from the self-reported BASIS-24 was used for measuring difficulties in interpersonal relationships. The subscale contains questions about how the person (1) gets along with people inside the family, (2) outside the family, (3) gets along in social situations, (4) feels close to another person, and (5) feels there is someone to turn to if help is needed [66]. The 5-items on this subscale are answered on a Likert scale ranging from zero (all the time) to four (none of the time). The scores were recoded and calculated as described in the BASIS-24 instruction guide [67], providing a score between 0 and 4 with higher scores indicating more severe problems. The BASIS-24 scale has demonstrated good construct validity and internal consistency [66, 68, 69].

#### Secondary outcomes for patients

##### Behavior and symptom identification scale (BASIS-24)

The self-reported BASIS-24 was used to measure the general experience of mental health and functioning [66]. BASIS-24 is a 24-item self-reported questionnaire distributed on six subscales: depression/functioning, emotional lability, self-harm, relationships (primary outcome), substance abuse, and psychosis. Scores were calculated as outlined in the BASIS-24 instruction guide [67], yielding a range from zero to four, where higher scores indicate more severe issues. The BASIS-24 scale has demonstrated good construct validity and internal consistency [66, 68, 69].

##### Overall perceived mental health burden

One single self-reported question of overall perceived mental health burden was made for the IFIP trial: “How troublesome do you think your mental health problems are at the moment?”, with scores ranging from one (very troublesome) to seven (not troublesome).

##### Perceived expressed emotions

The self-reported Perceived Criticism (PC) [70] and Perceived Warmth (PW) [71] were used to measure perceived expressed emotions from the nearest relative. The questionnaire was adapted in collaboration with Jill Hooley in 2019 to a five-item measure using a ten-point Likert scale ranging from one (not at all) to 10 (very) for each of the following questions: (1) How critical is (s)he of you?, (2) How warm is (s)he towards you? (3) How disapproving is (s)he of what you do?, (4) How caring is (s)he of you?, (5) When (s)he criticizes you, how upset do you get? The PC and PW are well-validated [72–74].

##### The recovering quality of life (ReQoL-10)

The self-reported ReQoL-10 was used to measure the outcomes of recovery in terms of those aspects of quality of life that matter to mental health service users [75]. It is a 10-item measure with scores ranging from zero (none of the time) to four (mostly, or all, of the time). In addition, ReQoL has one single question about physical health, with scores from zero (very severe problems) to four (no problems): “Please describe your physical health (problems with pain, mobility, difficulties caring for yourself or feeling physically unwell) over the last week”. ReQoL-10 has acceptable internal consistency and interrater reliability [75].

##### Manchester short assessment of quality of life (MANSA)

One question (number 10) from the self-reported MANSA [76] was used to measure overall quality of life: “How satisfied are you with your life as a whole today?”. The scale ranges from one (couldn’t be worse) to seven (couldn’t be better). The MANSA has good construct validity and internal consistency [76].

##### Health of the Nation outcome scale (HoNOS)

The clinician-rated HoNOS was used to measure overall functioning [77]. Twelve items in four domains (behavioral problems, organic problems, psychological symptoms, social problems), with scores ranging from zero (no problems) to four (severe problems), were summed. The scale has moderately high internal consistency and interrater reliability [77–79].

### Sample size calculations

The needed sample size was calculated based on an estimate related to the primary outcome, which was the Relationships subscale of BASIS-24. To detect an increase of 0.5 SD from baseline to 12 months, with 80% power and 5% two-tailed significance level, for a two-sided test, in a cRCT with an intra-class correlation coefficient (ICC) of 0.05, seven clusters in each group and a sample size of 112 patients was needed in each arm [55, 80, 81].

### Statistical analyses

The statistical methods used in this article closely resemble those in the relative outcomes sub-study [56] and are summarised below.

A chi-squared test compared the intervention and control arms with regard to number of patients possessing a crisis plan, and the types and extent of family involvement and support that patients and their relatives received from the CMHC units. Recruitment and inclusion were concurrent with the clinical interventions, meaning some relatives received services before inclusion, aligning with the inclusion criteria. Thus, we included “previous participation” in our results.

Means, standard deviations, and number of observations were calculated for all patient outcomes. As outlined in the study protocol [55], linear mixed-effects models estimated mean outcomes per treatment arm at baseline, 6 months, and 12 months. The model’s fixed part included intercept, treatment, time (dummy variables for 6 months and 12 months), and interaction terms between treatment arm and time variables. The random part comprised patient level intercept nested within centre level. When centre level variance was negligible, only patient level intercepts were included.

Patients with at least one follow-up measurement (6 or 12 months) were included, with missing data imputed using multiple imputations. This model incorporated age, sex, treatment arm, centre, and previous outcome scores, deploying predictive mean matching to create 30 data sets [82].

We reported the estimate of difference in change between intervention and control arms, 95% CI, Cohen’s d for effect sizes, and p-values. Regression coefficients, standard errors, 95% confidence intervals (CI), p-values, random effects variances, and goodness-of-fit through marginal and conditional R-squared were provided. Tests were two-sided with a 5% significance level, and residual plots were inspected graphically for model diagnostics. For exploratory secondary outcomes, adjustments for multiple testing were not applied presenting crude p-values interpretation. R version 3.4.4 (lmer-functions) was used for mixed-effects model estimations.

### Sensitivity analyses

We conducted a complete case analysis (subset of data with complete outcomes) and examined whether any differences from the main analysis on imputed data were consequential.

Since educational level and employment status for patients were found to be statistically significantly different between study arms at baseline, the main analysis described above was repeated with patient educational level and employment status included as fixed effect.

Baseline characteristics of the patients who were eliminated from the analysis due to missing outcomes at both 6 months and 12 months measurements were compared to the remaining patients with regard to age, sex, GAF, and HoNOS.

### Changes to the protocol

In response to the challenges we encountered during the project period, particularly regarding the analysis and publication of results for patients and relatives, we made three changes related to the statistical analysis plan in the protocol article [55]. First, the protocol article specifies that one of the secondary outcomes of the IFIP trial is to investigate whether a higher level of implementation of the selected recommendations for family involvement is associated with improved outcomes for patients and relatives. We decided to discontinue these analyses due to a lack of changes in the outcome measures, presumably largely as a result of the COVID-19 pandemic (please refer to the discussion of the findings). In this paper, we have confined our analyses to the differences in change over time between the intervention and control arms.

Second, the protocol article only defined fidelity as the primary outcome. The IFIP trial is a large and complex study with data collection and outcome measures at multiple levels: fidelity represents the implementation outcome measured at the health service level, whereas the patient and relative outcomes sub-studies are conducted at the individual level. After careful consideration and prior to conducting the analyses, we decided to define primary outcomes for both the patient and relative sub-studies. The IFIP trial was designed to ensure that both sub-studies had their own primary outcomes, reflected in the way the sample size calculations are presented in the protocol article. For the relative sub-study, satisfaction with health service support - measured by the Carer Well-being and Support Questionnaire Part B (CWS-B) - was used in the sample size calculation and subsequently defined as the primary outcome. For the current sub-study, the Relationships subscale from BASIS-24 was used to calculate the sample size and subsequently used as the primary outcome [55].

Third, the inclusion criteria allowed participants to have already received some level of FPE before baseline, and fewer individuals than expected were offered FPE during the data collection period. We therefore assumed that the effect of the FIs, implemented through the ISP, had been weakened. Therefore, we wanted to examine the outcome differences for a subgroup with stricter inclusion criteria, which included patients in the treatment arm who received FPE during the intervention period and excluded patients in both arms who had received FPE before baseline. As in the relative outcomes sub-study [56], we compared: (1) Patients in the intervention arm who received FPE during the 12 months following inclusion (*N* = 29), (2) Patients in the control arm who did not receive FPE during the 12 months prior to inclusion (*N* = 94). The estimated model included the same effects as the main linear mixed-effects models described above, in addition to the patient’s duration of psychotic disorder, the patient’s GAF-F, the relative’s marital status, whether the relative had higher education, and whether the relative resided with the patient. We anticipated larger effect sizes in the sub-group analyses. Due to the disparity in patient numbers between the groups and with a low total sample in the subgroups, we did not expect statistically significant differences between the groups.

## Results

### Participant flowchart

**Fig. 2:**
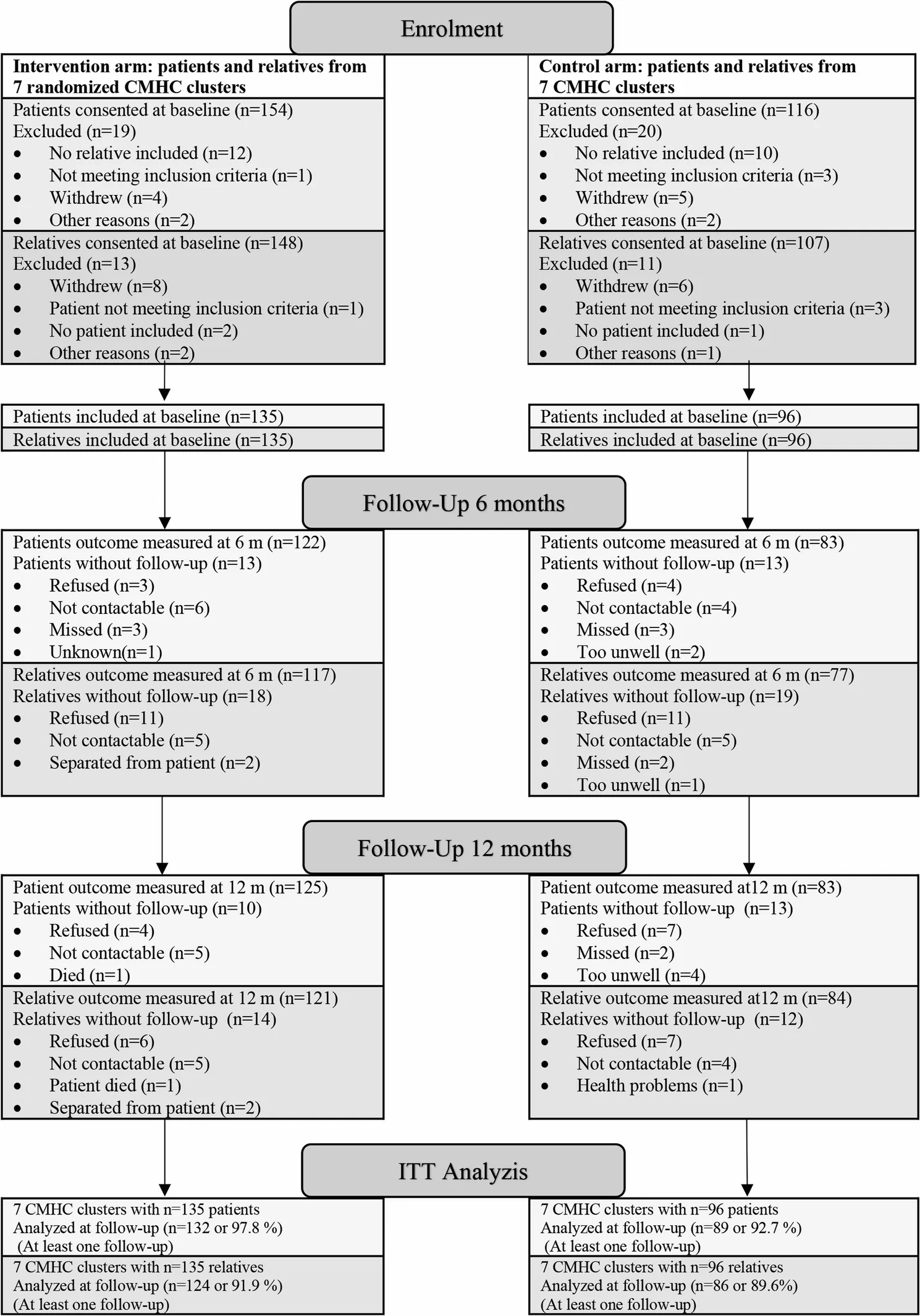
Data in this figure are republished from the article Implementation of guidelines on Family Involvement for persons with Psychotic disorders: a pragmatic cluster randomized trial. Effect on relatives’outcomes and family interventions received fpsyt-15-1381007-g001.jpg (2008×2869)

Figure 2 shows the previously published participant flowchart [56]. In total, 270 patients (154 intervention; 116 control) and 255 relatives (148 intervention; 107 control) from the 14 randomised clusters consented to the trial. This paper presents the analyses concerning the patients in the (complete) 231 patients/relative pairs (135 intervention; 96 control). Except for the first 24% of the 6 months’ questionnaires, the patients’ 6 months and 12 months data were collected during the COVID-19 pandemic.

### Baseline characteristics

Table 2 shows the sociodemographic characteristics and patient-reported adherence to prescribed medication. Most patients had a recurrent psychotic disorder, with an average duration of nine years. The mean GAF scores were almost identical in both arms at baseline (Intervention: GAF-S = 52.57 (SD = 13.21), GAF-F = 51.54 (SD = 13.04), TAU: GAF-S = 52.18 (SD = 10.65), GAF-F = 51.45 (SD = 12.06)).

The only significant differences in sociodemographic variables between the study arms were the patients’ educational level and main activities, with a higher proportion of patients in the intervention arm holding higher education qualifications and being in employment.

**Table 2:** Sociodemographic characteristics for patients at baseline

Characteristics	Total (*n* = 231)	Intervention (*n* = 135)	Control (*n* = 96)	*p*-value^a^
Age				0.30
	35.95 ± 12.55	35.1 ± 11.4	36.8 ± 13.7	
Gender				0.75
Male	134 (58)	80 (59.3)	54 (56.3)	
Female	97 (42)	55 (40.7)	42 (43.8)	
Place of birth				0.42
Norway	197 (85.3)	113 (83.7)	84 (87.5)	
Abroad	34 (14.7)	22 (16.3)	12 (12.5)	
Educational level		(3 missing)		0.023
Primary school	82 (36)	50 (37.9)	32 (33.3)	
Secondary school	97 (42.5)	47 (35.6)	50 (52.1)	
Higher education	49 (21.5)	35 (26.5)	14 (14.6)	
Civil status				0.28
Single	169 (73.2)	95 (70.4)	74 (77.1)	
Married/cohabiting	45 (19.5)	31 (23.0)	14 (14.6)	
Divorced, separated, widowed	17 (7.3)	9 (6.7)	8 (7.1)	
Living arrangement		(1 missing)	(1 missing)	0.96
Alone	127 (55.5)	75 (56.0)	52 (54.7)	
With someone else	102 (44.5)	59 (44.0)	43 (45.3)	
Caring for children		(1 missing)		0.83
Yes	19 (8.3)	12 (9.0)	7 (7.3)	
Main activity the last 6 month				0.003
Unemployed	123 (53.3)	75 (55.6)	48 (50)	
Employed	22 (9.5)	19 (14.1)	3 (3.1)	
Other	86 (37.2)	41 (30.4)	45 (46.9)	
Age when diagnosed				0.44
	27.1 ± (8.8)	26.6 ± 7.4	27.5 ± 10.2	
Diagnosis				0.22
Core schizophrenia (F20,21,22,25)	192 (83.1)	109 (80.7)	83 (86.5)	
Acute and transient psychosis (F22)	18 (7.8)	10 (7.4)	8 (8.3)	
Other psychosis (F28, 29)	21 (9.1)	16 (11.9)	5 (5.2)	
Adherence to medication^b^			(2 missing)	0.89
Yes	209 (91.3)	124 (91.9)	85 (90.4)	

Data are n (%), mean ± SD. n, number of participants

*SD* Standard deviation

^a^Calculated with Chi-square test (χ^2^) or t-test

^b^Patient-reported adherence to prescribed medication. 7 (5.2 %) of the patients in the intervention arm and 7 (7.4 %) in the TAU arm did not take medication

### Effectiveness of the ISP on service level outcomes

To understand the results of this study at the patient level, we reiterate the percentage of patient-relative pairs participating in FPE from the relative outcomes sub-study [56]. In the intervention arm, FPE increased from 17.2% (23/134) at baseline to 37.9% (50/132) at 12 months, and in the control arm, from 3% (3/96) to 7% (6/86) (*p* < 0.05). The differences in baseline FPE primarily stemmed from the study design and timeline challenges [56]. The training and initiation of clinical supervision of FIs in the intervention arm coincided with the recruitment of patients and their relatives. Furthermore, the recruitment period was extended from 12 to 19 months, partly due to the COVID-19 pandemic. As a result, some patients and relatives in the intervention arm had already started FPE by the time they were included. By the 13th study month, following the pandemic’s onset, only six new FPE groups were established in the intervention arm and two in the control arm [56].

During the data collection period, the mean number of FPE sessions in the intervention arm was 10.56 (SD = 8.71) and 8.5 (SD = 5.43) in the control arm.

Table 3 presents the previously published descriptive analyses of various types of BFIS consultations the patients’ relatives received with and without the patient from the CMHC units [56].

**Table 3:** Various types of family involvement and support received by the relatives from the CMHC units

		Previous				24 months				Change previous vs. 24 months	Intervention vs. control	*p*-value Z-test
N relatives intervention (I) = 134* N relatives control (C) = 96		n	N	%	p-val Chi-sq	n	N	%	p-val Chi-sq	Percen-tage points	Percen-tage points	
Supportive/next of kin consultation without the patient	I	37	134	27.6		77	120	64.2		36.6		
	C	27	96	28.1	1	37	81	45.7	**0.014**	17.6	19.0	**0.04**
Supportive/next of kin consultation with the patient	I	38	134	28.4		87	121	72.0		43.5		
	C	21	96	21.9	0.338	44	83	53.0	**0.009**	31.1	12.4	0.165
Consultation with the patient’s clinician	I	32	134	23.9		99	128	77.3		53.5		
	C	23	96	24.0	1	56	85	65.9	0.092	41.9	11.5	0.176

Data in this table are republished from the article Implementation of guidelines on Family Involvement for persons with Psychotic disorders: a pragmatic cluster randomized trial. Effect on relatives’outcomes and family interventions received fpsyt-15-1381007-t002.jpg (1460×1232) (frontiersin.org)

Bold numbers: p-value ≤ 0.05

*excluding 1 missing at inclusion

The proportion of patients in the intervention arm who had a crisis plan increased from 60.9% (81/133) at baseline to 72.7% (96/132) at 12 months, and decreased in the control arm, from 67.7% (65/96) to 65.9% (56/85) (*p* = 0.133).

### Effectiveness of the family interventions, implemented through the ISP, on patients’ outcomes

Table 4 presents the average (mean, standard deviation) patient outcome measures at each time point, and the estimated difference in change between baseline and 6- and 12 months, which was the treatment effect. Supplementary File 2 presents the estimated within-arm changes in outcome from baseline to 6- and 12 months, as well as the treatment effect for completeness, including 95% confidence intervals, corresponding effect size (Cohen’s d) and p value. Supplementary File 3 presents the regression coefficients from the estimated linear mixed-effects models, the estimated random effects variances and ICC, as well as marginal and conditional R-squared for each outcome.

**Table 4:** Descriptive statistics for patient outcomes at baseline (T1), 6 month (T2), and 12 month (T3), and the estimated treatment effect from baseline to 6 and 12 months

			Control		Intervention	Treatment effect (95% CI)	Cohen’s d	*p*
		*n*	Mean (SD)	*n*	Mean (SD)			
BASIS-24 mean score^a^	T1	93	1.2 (0.75)	135	1.15 (0.66)			
	T2	81	1.1 (0.72)	119	1.04 (0.69)	0.01 (−0.15, 0.18)	0.02	0.88
	T3	83	1.08 (0.65)	124	1.01 (0.67)	−0.02 (−0.18, 0.14)	−0.03	0.81
Depression functioning^a^	T1	93	1.33 (0.96)	135	1.31(0.9)			
	T2	81	1.21(0.91)	120	1.19 (0.94)	0.06 (−0.18, 0.29)	0.06	0.64
	T3	83	1.15 (0.83)	124	1.11 (0.87)	0.01 (−0.22, 0.24)	0.01	0.91
Emotional lability^a^	T1	94	1.6 (0.9)	135	1.4 (0.85)			
	T2	81	1.49 (0.97)	121	1.24 (0.88)	−0.05 (−0.27, 0.18)	−0.05	0.69
	T3	83	1.47 (0.84)	124	1.2 (0.83)	−0.08 (−0.31, 0.15)	−0.09	0.49
Self harm^a^	T1	94	0.41 (0.75)	135	0.36 (0.77)			
	T2	81	0.33 (0.72)	121	0.35 (0.81)	0.09 (−0.1, 0.27)	0.12	0.37
	T3	83	0.33 (0.63)	124	0.37 (0.75)	0.08 (−0.1, 0.26)	0.11	0.39
**Relationships** ^a^	**T1**	**96**	**1.31 (0.97)**	**135**	**1.32 (0.85)**			
**(primary outcome)**	**T2**	**83**	**1.19 (0.85)**	**122**	**1.23 (0.88)**	**−0.03 (−0.29**,** 0.23)**	**−0.03**	**0.83**
	**T3**	**83**	**1.29 (0.97)**	**124**	**1.16 (0.82)**	**−0.17 (−0.43**,** 0.09)**	**−0.19**	**0.2**
Substance abuse^a^	T1	96	0.56 (0.96)	135	0.36 (0.63)			
	T2	83	0.33 (0.68)	122	0.29 (0.54)	0.08 (−0.07, 0.23)	0.11	0.29
	T3	83	0.51 (0.82)	125	0.33 (0.63)	−0.01 (−0.16, 0.14)	−0.01	0.93
Psychosis^a^	T1	94	1.02 (1.09)	135	0.93 (0.98)			
	T2	81	0.96 (1.06)	121	0.81 (1.01)	−0.07 (−0.29, 0.15)	−0.07	0.56
	T3	83	0.87 (0.9)	124	0.84 (1.05)	0.03 (−0.18, 0.25)	0.03	0.77
IFIP question burden^b^	T1	93	4.35 (1.83)	135	4.39 (1.97)			
	T2	81	4.83 (1.84)	121	4.97 (1.83)	−0.03 (−0.53, 0.47)	−0.02	0.91
	T3	83	4.61 (1.77)	123	4.95 (1.89)	0.33 (−0.17, 0.82)	0.17	0.19
1. PC^c^	T1	94	3.15 (2.41)	135	3.85 (2.55)			
	T2	81	3.21 (2.3)	120	3.38 (2.37)	−0.58 (−1.35, 0.18)	−0.25	0.14
	T3	83	3.6 (2.46)	124	3.32 (2.18)	−0.94 (−1.69, −0.2)	−0.4	0.01
2. PW^c^	T1	94	8.2 (2.28)	135	7.75 (2.14)			
	T2	81	8.32 (1.82)	120	7.42 (2.39)	−0.43 (−1.03, 0.18)	−0.19	0.16
	T3	83	7.88 (2.2)	124	7.14 (2.55)	−0.4 (−0.99, 0.2)	−0.17	0.19
3. PC^c^	T1	94	3.3 (2.71)	135	3.32 (2.3)			
	T2	81	3.02 (2.16)	120	3.14 (2.34)	−0.08 (−0.87, 0.7)	−0.03	0.84
	T3	82	3.15 (2.43)	124	3.19 (2.45)	−0.1 (−0.86, 0.67)	−0.04	0.8
4. PW^c^	T1	94	8.4 (2.21)	135	8.06 (2.18)			
	T2	81	8.26 (1.95)	120	7.77 (2.35)	−0.25 (−0.99, 0.48)	−0.11	0.5
	T3	82	8.1 (2.22)	124	7.94 (2.27)	0.04 (−0.68, 0.76)	0.02	0.91
5. PC^c^	T1	92	4.6 (2.62)	135	4.9 (2.84)			
	T2	80	4.24 (2.6)	121	5 (2.85)	0.47 (−0.37, 1.31)	0.18	0.28
	T3	81	4.64 (2.38)	123	4.84 (2.62)	−0.16 (−0.99, 0.67)	−0.06	0.7
ReQoL sum score^d^	T1	96	24.08 (8.3)1	135	24.26 (8.17)			
	T2	83	25.8 (7.53)	121	24.84 (8.41)	−1.06 (−3.12, 1)	−0.13	0.31
	T3	81	25.84 (8.22)	124	26.35 (8.33)	0.48 (−1.6, 2.56)	0.06	0.65
ReQoL physical health^d^	T1	92	2.93 (1.03)	135	2.97 (0.97)			
	T2	81	3.06 (0.95)	122	3.14 (0.87)	0.06 (−0.2, 0.32)	0.07	0.63
	T3	83	2.98 (1.02)	123	3.19 (0.83)	0.22 (−0.04, 0.48)	0.24	0.1
MANSA^e^	T1	93	4.49 (1.35)	135	4.48 (1.41)			
	T2	81	4.57 (1.38)	122	4.57 (1.5)	0.09 (−0.34, 0.53)	0.06	0.68
	T3	83	4.58 (1.47)	123	4.51 (1.4)	−0.02 (−0.45, 0.4)	−0.02	0.91
HoNOS sum score^f^	T1	95	8.91 (5.58)	133	9.05 (5.32)			
	T2	0	NA	0	NA	NA	NA	NA
	T3	83	9.28 (7.03)	127	8.13 (6.03)	−1.73 (−3.05, −0.42)	−0.29	0.01

Presented descriptive statistics are Mean (SD). Primary outcome: bold numbers

^a^The Behavior and Symptom Identification Scale (BASIS-24)

^b^IFIP trial question: Experienced burden of mental health problems

^c^Perceived criticism (PC) and perceived warmth (PW) from relative: 1) How critical is he/she of you, 2) How warm is he/she towards you, 3) How disapproving is he/she of what you do?, 4) How caring is he/she of you?, 5) When he/she criticizes you, how upset do you get?

^d^The Recovering Quality of Life questionnaire (ReQoL-10)

^e^The Manchester Short Assessment of Quality of Life (MANSA). One question: How satisfied are you with your life as a whole today?

^f^The Health of the Nation Outcome Scale (HoNOS)

### Primary outcome

There were no significant differences in patients’ difficulties in interpersonal relationships between the intervention and control arms at 12 months (Cohen’s d = −0.19, *p* = 0.2). The ICC for this outcome was 0.044 (CI = 0.042, 0.045).

### Secondary outcomes

Patients in the intervention arm perceived less criticism from the relatives (PC sub-score; d = − 0.4, *p* = 0.01) compared to the control arm at 12 months. In both arms patients perceived a reduction in warmth (PW) from relatives at 12 months (no significant differences).

Reduced difficulties in the patient’s overall functioning (HoNOS; d = − 0.29, *p* = 0.01) were seen in the intervention arm compared to the control arm at 12 months. A slight trend in decreased mental health and functioning problems (most subscales of BASIS-24 and the IFIP question on symptom burden), and in increased recovery and quality of life (ReQoL, MANSA) were experienced in both arms, from 0 to 12 months, but all differences were non-significant.

### Subgroup analysis

In the subgroup analysis, we compared patients in the treatment arm who only received FPE during the 12 months after inclusion, with patients in the control arm who did not receive FPE the last 12 months before inclusion (data not shown). For the primary outcome, difficulties in interpersonal relationships (BASIS-24 subscale), we found that the treatment effect was in the same direction and showed the same positive trend as in the main analysis, but with a larger effect size. Regarding the secondary outcomes, the treatment effect for difficulties in overall functioning (HoNOS) was consistent in direction and exhibited similar positive trends to the main analysis, but with a greater effect size. In the subgroup analysis for perceived criticism (PCM sub-score) results revealed a continuation of the positive trend in the main analysis, indicating reduced criticism, though with a diminished effect size. As expected, no significant changes from baseline to 12 months were observed between the two groups.

### Sensitivity analyses

Regression coefficients from the complete case analysis on patient outcomes (data not shown) were in the same direction and of similar magnitude as the results from the main analysis described above. Further, the sensitivity analyses where either educational level or employment status for patients were included as a fixed effect, did not yield significantly different results for the estimated treatment effect (data not shown).

When the baseline characteristics (age, sex, GAF, and HoNOS) of the participants who were lost to 6 months and 12 month measurements were compared to the included patients, it was found that patients in the control arm with no follow-up exhibited higher scores (indicating more problems) on the HoNOS (*N* = 13, mean = 11.15, SD = 7.01) than those with at least one follow-up (*N* = 83, mean = 8.55, SD = 5.29, *p*=0.22).

## Discussion

The current sub-study aimed to examine the effectiveness of FIs implemented through the ISP, in comparison to TAU, on patient outcomes. The ISP, which facilitated the FIs, increased clinicians’ family involvement significantly. However, there was no significant effect on the patients’ perceived interpersonal relationships. The impact on secondary outcomes, including decreased criticism from relatives and enhanced overall functioning, remains ambiguous. This uncertainty arises from the numerous exploratory analyses conducted, which may elevate the likelihood of false positive findings. Earlier RCTs of FIs have focused on interventions at the level of individuals and a strictly defined patient population [20]. This naturalistic study assesses the effectiveness of FIs implemented through the ISP among a highly diverse patient population at the CMHC units, and where not all patients received FIs, and the duration and timing of FIs received varied. Furthermore, some patients in the control arm received FIs. Consequently, the findings cannot be directly compared to previous clinical trials.

There was a substantial increase in the proportion of included patients receiving both BFIS and FPE in the intervention arm [56]. However, the increase in FPE and number of sessions throughout the 12 months observation period were less than anticipated. The increase in FPE resembles two studies in which FPE was systematically offered as part of an integrated treatment program to patients with a first episode psychosis. In the OPUS-study 48.8% of the patients participated in a multi-family group or a single-family group [83], and in the TIPS project 59.8% of the patients participated in a multi-family group [84]. In our study, the proportion of FPE was lower (37.9%). This might be due to the pandemic and the study design, but also the population, the proportion of clinicians with FPE competence and capacity, and differences in the intervention; such as BFIS to all patients, while FPE in single-family groups to as many as possible [56, 57].

The FIs implemented through the ISP led to more patients having a crisis plan in the intervention arm compared to the control arm. The relative outcomes sub-study found increased involvement of family members in these plans [56]. Crisis plans have been shown to provide support and reassurance during crises, crucial for enhancing patients’ mental health and minimising crises occurrences [85–87].

For the primary outcome, we observed a reduction in patient-reported difficulties in interpersonal relationships (BASIS-24 subscale) in both arms, with the largest reduction occurring in the intervention arm. The subgroup analysis also showed reductions but with a larger effect size. Contrary to what we had expected, neither of the differences were significant.

One potential reason for not observing a significant reduction in difficulties with interpersonal relationships could be the lower-than-expected number of patients receiving FPE and the lower-than-expected quantity of FPE provided after the baseline measurement. Both were interrupted by the COVID-19 pandemic. BFIS alone may not suffice to address these challenges. The pandemic restricted social interaction, with the Norwegian Government advising people to avoid contact with others [60]. Most participants in a Norwegian study involving individuals with psychosis and bipolar disorder reported a decline in their social life, feeling increasingly isolated, excluded, and lonely following the outbreak. Some also experienced an exacerbation of family conflicts [88]. Alterations in social relationships may require a considerable amount of time, and the pandemic reduced patients’ opportunities to utilize the acquired communication skills obtained through FPE and to improve interpersonal relationships. Additionally, all participants scored low on interpersonal relationships difficulties, which may have limited the ability to demonstrate the effectiveness of FIs through ISP due to a ceiling effect, leaving little room for improvement. We also acknowledge that the choice of the Relationship subscale of BASIS-24 as the primary outcome may not be the most optimal measure for assessing the effects of FIs. Only three out of five questions (question 1, 4, and 5) could be regarded as directly related to interpersonal relationships within the family. Together, these factors might have contributed to an underestimation of the reduction in difficulties in interpersonal relationships in the intervention arm.

The IFIP trial included qualitative studies with selected patients [89] and clinicians [90] from the intervention arm involved in FIs. These studies revealed that FPE could enhance familial relationships by fostering conversations that promote mutual understanding, a sense of ‘being on the same team’, and better communication and problem-solving skills in daily life [89, 90].

For the secondary outcomes, the difference in the patients’ perceived criticism (PC) from their relatives was a combined result from increased criticism in the control arm and decreased criticism in the intervention arm. The increased support received through FIs may have served not only to reduce criticism but also to alleviate relatives’ stress related to accessing help for patients during the pandemic [91]. Conversely, the pandemic may have intensified relatives’ concerns for the patients’ health [88] in the control arm, leading to higher criticism level.

Subgroup analyses indicated a similar but smaller effect on perceived criticism, suggesting that BFIS, which improves relatives’ understanding of the patient’s situation [28, 92], sufficiently reduced patients’ perceived criticism from relatives.

The qualitative patient study confirmed the mentioned interpretation, by describing how the relatives’ increased knowledge led to a relational shift, including reduced criticism [89]. The qualitative clinician study revealed that FIs reduced conflict and stress by improving contact, cooperation, and openness among patients, relatives, and health personnel. The results were described as ‘lowered shoulders,’ ‘calmer relatives and home environment,’ and ‘reduced nagging and ‘critical comments’ [90]. Perceived criticism from the perspective of the patient, is a valid contributing predictor of clinical outcomes [93] and has been linked to a poorer illness course in individuals with schizophrenia [94, 95]. Family interventions targeting high levels of the negative aspects of expressed emotions (criticism, hostility, emotional overinvolvement) in families, have been repeatedly shown to improve long-term outcomes in persons with psychotic disorders in general [20] and in early psychosis [30, 96].

Patients in the intervention arm showed a reduction in difficulties with overall functioning (HoNOS) while those in the control arm experienced an increase. Subgroup findings also indicated a positive trend, with a larger effect size. However, the standard deviation of HoNOS is large, and the effect size is small. It is also worth noting the large variation in age, life situation, and functioning. Additionally, there may have been variations in the course of the illness and in the characteristics and effects of the treatment that patients were provided routinely. The diversity of the patient group, comprising both first episode and long-term psychosis, led to a variety of potentials for improvement during the measurement period. Interpreting the reduction in patients’ difficulties with overall functioning should be done with caution. However, substantial research has found positive effects of FIs on patients’ global functioning [11, 12, 19].

The FIs, implemented through the ISP, may also have relieved the relatives’ responsibility and caregiver burden [56]. Our findings show that the intervention promoted collaboration among family, patients, and clinicians [90]. Many patients in our qualitative patient study reported enhanced support from relatives due to FIs, and some experienced improved problem-solving in everyday life and relapse prevention [89]. The relative outcomes sub-study indicated that greater support through FIs, including crisis planning involvement, could reduce perceived patient dependency [56]. Collectively, these factors may have fostered a better family environment and supported the patient’s journey towards improved functioning [97–99].

### Box 1. Commentary: Lived experiences by Solveig H. H. Kjus

I have personal experience participating in a psychoeducational multi-family group (MFG) from 2008 to 2010. The MFG has continued as a self-help group, and we have now been meeting regularly for 15 years. The group has become a valuable part of my network. I have commented on drafts of this article and participated in the advisory board of the IFIP trial.

The current sub-study finds that participants receiving FIs perceived less criticism from their relatives. Even though I did not experience direct criticism from my family members before I joined the multi-family group, we were not very good at talking about feelings. It was also difficult that my family members did not understand my problems. Through the group, we developed a common language to talk about emotions. This made communication easier and our relationship even better. Just the fact that my family members prioritized participating in the group felt like proof that they cared about me. We simply grew closer as a family. It was also safer to share how I was feeling in the group than alone with my family members. Before the group, I was afraid that my family members would become scared or behave differently towards me if I told them, for example, that I was having a bad day. In the group, I could tell them what it truly feels like to struggle with this condition because I had more people involved in the conversation, and I felt that both I and my family members had the support we needed around us. My family members thereby gained increased knowledge and a greater understanding of what I was going through. This was of great help to all of us.

In the group meetings, we focused on solutions to both small and large challenges, which made it possible for me to accomplish things that were important to me. These could be strategies for how I could handle social situations with friends and family, such as Christmas gatherings. Over time, I experienced better functioning in everyday life. It also felt very meaningful to contribute suggestions for solutions to the other participants in the group.

We also worked on crisis management. In acute crisis, I received good help from the group leaders in contacting the urgent care/ambulance after I had managed to inform them. This made it possible for me to get help at an earlier stage of crisis than before, resulting in less suffering and not so dramatic admissions.

### Consideration

The IFIP study implemented a comprehensive and complex intervention at the level of CMHC units, covering a vulnerable patient population across multiple sites in a real-world setting. The patients in the current sub-study were recruited regardless of their assignment to FIs [55]. All 7 units in the intervention arm encountered various issues that complicated or hindered patients from participating in FIs, reflecting the realities of routine clinical practice.

Previous studies of FIs have shown that FIs should preferably include the patient and have a duration of at least 9 months to influence well-being and health [100]. The current sub-study assessed the effectiveness of the FIs implemented through the ISP over a 12-months period in a naturalistic setting. The proportion of patients receiving FIs was lower than expected, and both the duration and timing of receiving FIs varied. Despite the limited number of significant findings - likely due to the constrained implementation of FIs in the intervention arm after baseline measurement, uncontrollable external disturbances, and a diverse patient population - the results of this sub-study still provide valuable evidence about FIs for individuals with psychotic disorders within routine mental health services. This evidence is highly needed [101, 102].

### Strengths and limitations

The particular strength of this sub-study lies in its large, representative sample of patients who are seldom targeted by intervention studies, alongside notably high response rates at 6 months and 12 months. With regard to the analyses, a strength is that the subgroup analyses supported the main findings concerning patients’ positive outcomes.

 The pragmatic naturalistic design, which considers a wide range of patient-centred outcomes, provides high generalizability and clinical value for CMHC units offering FIs [103]. However, exploratory analyses of numerous outcomes increase the potential for false positive findings.

The hybrid effectiveness-implementation cluster randomised design has weakened the measured effectiveness of the IFIP study among patients. One of the main limitations of the sub-study is the variability and low level of therapeutic doses of FIs within the intervention arm after the baseline measurement, as well as the fact that participants in the control arm also received some degree of FIs [56]. The inconsistent implementation of FIs across participating CMHC units underscores both the real-world challenges associated with guideline implementation and the challenges in demonstrating treatment effects in naturalistic studies.

The IFIP trial did not achieve the expected sample size for the control arm, as determined by the power analysis. This may be another reason why the results did not reach statistical significance, particularly considering the small effect sizes. In addition, unequal allocation of patient numbers across the clusters in the CMHC units may have led to reduced statistical power, skewing of overall results and bias, which could have affected the estimation of the ISP effect on patient outcomes and potentially limited the generalisability of our findings.

Further limitations are presented and discussed previously in the relative outcomes article [56] and include the following factors: (1) Patients’ inclusion criteria; broad criteria, some level of FPE received before inclusion were accepted, a highly diverse patient group, (2) Existing family involvement; a lot in both arms at baseline and afterwards, FIs implemented through the ISP as an add-on intervention, (3) Study design and timeline challenges; recruitment of patients and training in the clinical interventions at the same time, prolonged recruitment period due to the COVID-19 pandemic, limited resources, and a relatively short project period to provide BFIS and FPE to all patients, (4) Context: The COVID-19 pandemic affected both the health care services, the patients, and the ISP, consequently significantly disrupting the delivery of FIs.

### Implications

The current sub-study significantly enhances our knowledge about the potential challenges, as well as the positive impact on patient outcomes, of FIs implemented at the level of CMHC units. At baseline, only 4% of patients in the participating CMHC units had received FPE [46]. The IFIP trial is particularly clinically relevant and important by highlighting how an ISP can increase FIs in ordinary mental health practice.

The results of the IFIP trial should be leveraged to systematically increase the amount and improve the quality of family involvement practices for individuals with psychotic disorders in CMHCs. It is essential for mental health services to ensure a family-inclusive culture based on expertise and procedures to offer FIs to all patients and their relatives, adjusted to align with each family’s needs [104]. Our qualitative study on barriers and facilitators when implementing family involvement highlights barriers at the organisational level (e.g. lack of routines, resources, and logistics) and at the clinical level (e.g. factors related to patients, relatives, and clinicians) [47]. This aligns with the extensive research literature identifying several significant system-level barriers [45, 48, 105–107], as well as organisational cultures that may impede the uptake of family interventions [45, 105, 106]. Additionally, clinical obstacles include patients’ reluctance to involve their relatives and concerns regarding confidentiality [106–108], coupled with therapists’ lack of knowledge, competence and confidence [106]. Understanding these barriers could provide important insights for a potential replication study.

For future studies, ensuring statistical power by reaching the expected caseload number would increase the likelihood of detecting significant differences when they exist, thereby improving the robustness and reliability of the study findings. Additionally, the sub-study can contribute to the development and research on FIs for other patient groups in ordinary mental health services.

## Conclusions

Increased support and collaboration through FIs may lead to improved knowledge and reduced caregiver burden for the relatives, fostering a more positive family environment. This, combined with better crisis management, may enhance the patient’s progress toward improved functioning. As described in the relatives’ sub-study, the amount of FIs implemented through the ISP increased substantially. However, the amount of FIs did not significantly reduce patients’ difficulties in interpersonal relationships. However, patients perceived less criticism from relatives and reduced difficulties in overall functioning. Interpreting these results should be done with caution due to multiple testing.

The complexity of the study design, and the restrictions due to COVID-19, may have diminished the implementation and effectiveness of the measured FIs for the patients.

## Supplementary Information

Supplementary Material 1.

Supplementary Material 2.

Supplementary Material 3.

## Data Availability

The datasets used and analysed during the current sub-study are available from the corresponding author upon reasonable request.

## Abbreviations

IFIP: The Implementation of guidelines on Family Involvement for persons with Psychotic disorders.
CMHC: Community Mental Health Centre.
FIs: Family Interventions.
FPE: Family Psychoeducation.
BFIS: Basic Family Intervention and Support.
COVID-19: Coronavirus disease 2019.
CONSORT: Consolidated Standards of Reporting Trials.
TAU: Treatment as usual.
BASIS-24: The Behaviour and symptom identification scale.
HoNOS: Health of the Nation Outcome Scale.
PC: Perceived Criticism.
PW: Perceived Warmth.
ReQoL-10: The Recovering Quality of Life.
MANSA: Manchester Short Assessment of Quality of Life.
ICC: Intra-class correlation coefficient.
