# Supplementary material for: Effectiveness of implementing family involvement on patient outcomes in individuals with psychotic disorders: a pragmatic cluster randomised controlled trial
Source: BMC Psychiatry. 2025 Dec 4;25:1142. doi: 10.1186/s12888-025-07501-z (PMC12676765; doi:10.1186/s12888-025-07501-z)
Supplement: Supplementary file 2 — Supplementary Material 2. [file 12888_2025_7501_MOESM2_ESM.pdf]

Supplementary File 2. Estimated change in outcome and difference in outcome change between arms from baseline to 6 months and 12 months, 95% confidence intervals, corresponding effect size (Cohen's d) and p value

Outcome	6 MONTHS					12 MONTHS				
	0 to 6 m change	95% CI lower	95% CI upper	Cohen's d	P-value	0 to 12 m change	95% CI lower	95% CI upper	Cohen's d	P-value
BASIS-24 mean score Control	-0,11	-0,23	0,02	-0,16	0,10	-0,11	-0,23	0,02	-0,16	0,09
BASIS-24 mean score Intervention	-0,09	-0,20	0,01	-0,14	0,08	-0,13	-0,23	-0,03	-0,19	0,01
BASIS-24 mean score Difference	0,01	-0,15	0,18	0,02	0,88	-0,02	-0,18	0,14	-0,03	0,81
BASIS-24 Depression functioning Control	-0,15	-0,33	0,03	-0,17	0,10	-0,19	-0,37	-0,01	-0,21	0,04
BASIS-24 Depression functioning Intervention	-0,10	-0,24	0,05	-0,11	0,19	-0,18	-0,32	-0,03	-0,20	0,02
BASIS-24 Depression functioning Difference	0,06	-0,18	0,29	0,06	0,64	0,01	-0,22	0,24	0,01	0,91
BASIS-24 Emotional lability Control	-0,11	-0,28	0,07	-0,12	0,24	-0,11	-0,29	0,06	-0,13	0,20
BASIS-24 Emotional lability Intervetion	-0,15	-0,30	-0,01	-0,17	0,04	-0,19	-0,34	-0,05	-0,22	0,01
BASIS-24 Emotional lability Difference	-0,05	-0,27	0,18	-0,05	0,69	-0,08	-0,31	0,15	-0,09	0,49
BASIS-24 Self harm Control	-0,08	-0,23	0,06	-0,11	0,26	-0,09	-0,23	0,06	-0,12	0,23
BASIS-24 Self harm Intervetion	0,00	-0,12	0,12	0,00	0,96	-0,01	-0,12	0,11	-0,01	0,92
BASIS-24 Self harm Difference	0,09	-0,10	0,27	0,12	0,37	0,08	-0,10	0,26	0,11	0,39
BASIS-24 Relationships Control	-0,07	-0,27	0,13	-0,08	0,50	0,05	-0,15	0,25	0,06	0,60
BASIS-24 Relationships Intervetion	-0,10	-0,26	0,06	-0,11	0,24	-0,11	-0,28	0,05	-0,13	0,18
BASIS-24 Relationships Difference	-0,03	-0,29	0,23	-0,03	0,83	-0,17	-0,43	0,09	-0,19	0,20
BASIS-24 Substance abuse Control	-0,17	-0,29	-0,06	-0,25	0,00	-0,03	-0,14	0,09	-0,04	0,66
BASIS-24 Substance abuse Intervetion	-0,09	-0,18	0,00	-0,13	0,05	-0,03	-0,13	0,06	-0,05	0,49
BASIS-24 Substance abuse Difference	0,08	-0,07	0,23	0,11	0,29	-0,01	-0,16	0,14	-0,01	0,93
BASIS-24 Psychosis Control	-0,06	-0,22	0,11	-0,06	0,52	-0,14	-0,30	0,03	-0,14	0,11
BASIS-24 Psychosis Intervention	-0,12	-0,26	0,02	-0,12	0,09	-0,10	-0,24	0,03	-0,10	0,14
BASIS-24 Psychosis Difference	-0,07	-0,29	0,15	-0,07	0,56	0,03	-0,18	0,25	0,03	0,77
IFIP question burden Control	0,49	0,10	0,88	0,26	0,01	0,22	-0,17	0,60	0,12	0,27
IFIP question burden Intervention	0,46	0,15	0,78	0,25	0,00	0,54	0,23	0,85	0,29	0,00
IFIP question burden Difference	-0,03	-0,53	0,47	-0,02	0,91	0,33	-0,17	0,82	0,17	0,19
1. Perceived criticism Control	0,14	-0,45	0,73	0,06	0,64	0,51	-0,07	1,10	0,22	0,08
1. Perceived criticism Intervention	-0,44	-0,92	0,04	-0,19	0,07	-0,43	-0,90	0,04	-0,18	0,07
1. Perceived criticism Difference	-0,58	-1,35	0,18	-0,25	0,14	-0,94	-1,69	-0,20	-0,40	0,01
2. Perceived warmth Control	0,16	-0,30	0,63	0,07	0,49	-0,22	-0,68	0,24	-0,10	0,35
2. Perceived warmth Intervention	-0,26	-0,64	0,12	-0,12	0,17	-0,62	-1,00	-0,23	-0,27	0,00
2. Perceived warmth Difference	-0,43	-1,03	0,18	-0,19	0,16	-0,40	-0,99	0,20	-0,17	0,19
3. Perceived criticism Control	-0,14	-0,75	0,46	-0,06	0,65	-0,08	-0,67	0,51	-0,03	0,80
3. Perceived criticism Intervention	-0,22	-0,72	0,27	-0,09	0,37	-0,17	-0,65	0,31	-0,07	0,48
3. Perceived criticism Difference	-0,08	-0,87	0,70	-0,03	0,84	-0,10	-0,86	0,67	-0,04	0,80

Outcome	6 MONTHS					12 MONTHS				
	**0 to 6 m change**	**95% CI lower**	**95% CI upper**	**Cohen's d**	**P-value**	**0 to 12 m change**	**95% CI lower**	**95% CI upper**	**Cohen's d**	**P-value**
4. Perceived warmth Control	-0,02	-0,58	0,54	-0,01	0,95	-0,16	-0,72	0,40	-0,07	0,57
4. Perceived warmth Intervention	-0,27	-0,74	0,19	-0,12	0,25	-0,12	-0,57	0,34	-0,05	0,61
4. Perceived warmth Difference	-0,25	-0,99	0,48	-0,11	0,50	0,04	-0,68	0,76	0,02	0,91
5. Perceived criticism Control	-0,36	-1,02	0,30	-0,13	0,28	0,02	-0,63	0,66	0,01	0,96
5. Perceived criticism Intervention	0,11	-0,42	0,64	0,04	0,69	-0,15	-0,67	0,37	-0,06	0,58
5. Perceived criticism Difference	0,47	-0,37	1,31	0,18	0,28	-0,16	-0,99	0,67	-0,06	0,70
ReQoL sumscore Control	1,68	0,09	3,27	0,20	0,04	1,54	-0,10	3,17	0,19	0,07
ReQoL sumscore Intervention	0,62	-0,70	1,93	0,08	0,36	2,02	0,72	3,31	0,25	0,00
ReQoL sumscore Difference	-1,06	-3,12	1,00	-0,13	0,31	0,48	-1,60	2,56	0,06	0,65
ReQoL physical health Control	0,09	-0,12	0,29	0,09	0,40	-0,01	-0,21	0,19	-0,01	0,92
ReQoL physical health Intervention	0,15	-0,01	0,32	0,16	0,07	0,21	0,05	0,37	0,23	0,01
ReQoL physical health Difference	0,06	-0,20	0,32	0,07	0,63	0,22	-0,04	0,48	0,24	0,10
MANSA Control	0,02	-0,31	0,35	0,01	0,91	0,04	-0,29	0,37	0,03	0,80
MANSA Intervention	0,11	-0,15	0,38	0,08	0,41	0,02	-0,25	0,29	0,01	0,89
MANSA Difference	0,09	-0,34	0,53	0,06	0,68	-0,02	-0,45	0,40	-0,02	0,91
HoNOS sum score Control	NA	NA	NA	NA	NA	0,68	-0,34	1,71	0,11	0,19
HoNOS sum score Intervention	NA	NA	NA	NA	NA	-1,05	-1,87	-0,23	-0,17	0,01
HoNOS sum score Difference	NA	NA	NA	NA	NA	-1,73	-3,05	-0,42	-0,29	0,01

Abbreviations: Basis-24: The Behavior and Symptom Identification scale; IFIP question burden: Experienced burden of mental health problems; Perceived criticism and perceived warmth from relative: 1. How critical is he/she of you, 2. How warm is he/she towards you, 3. How disapproving is he/she of what you do?, 4. How caring is he/she of you?, 5. When he/she criticizes you. how upset do you get?; ReQoL-10: The Recovering Quality of Life questionnaire; MANSA: The Manchester Short Assessment of Quality of Life. One question: How satisfied are you with your life as a whole today?; HoNOS: The Health of the Nation Outcome Scale
