# Supplementary material for: Effectiveness of implementing family involvement on patient outcomes in individuals with psychotic disorders: a pragmatic cluster randomised controlled trial
Source: BMC Psychiatry. 2025 Dec 4;25:1142. doi: 10.1186/s12888-025-07501-z (PMC12676765; doi:10.1186/s12888-025-07501-z)
Supplement: Supplementary file 3 — Supplementary Material 3. [file 12888_2025_7501_MOESM3_ESM.pdf]

Supplementary File 3
Regression coefficients for all outcomes

Outcome	Parameter	Estimate	Standard error	95% CI lower	95% CI upper	p value
BASIS-24 mean score[a]	Intercept	1,181	0,073	1,038	1,325	<0.01
	Arm at baseline	-0,038	0,094	-0,223	0,147	0,687
	Time 6mo	-0,107	0,065	-0,233	0,02	0,1
	Time 12mo	-0,107	0,064	-0,231	0,018	0,093
	Time 6mo x Arm	0,013	0,083	-0,149	0,175	0,875
	Time 12mo x Arm	-0,02	0,082	-0,18	0,141	0,81
Depression functioning[a]	Intercept	1,324	0,097	1,134	1,514	<0.01
	Arm at baseline	-0,029	0,125	-0,273	0,216	0,818
	Time 6mo	-0,152	0,091	-0,332	0,027	0,095
	Time 12mo	-0,192	0,092	-0,372	-0,012	0,037
	Time 6mo x Arm	0,055	0,119	-0,177	0,288	0,641
	Time 12mo x Arm	0,013	0,118	-0,218	0,244	0,914
Emotional lability[a]	Intercept	1,568	0,093	1,386	1,75	<0.01
	Arm at baseline	-0,17	0,12	-0,405	0,064	0,155
	Time 6mo	-0,105	0,09	-0,282	0,072	0,244
	Time 12mo	-0,114	0,089	-0,289	0,06	0,199
	Time 6mo x Arm	-0,046	0,116	-0,274	0,182	0,691
	Time 12mo x Arm	-0,08	0,116	-0,308	0,148	0,49
Self harm[a]	Intercept	0,401	0,079	0,246	0,557	<0.01
	Arm at baseline	-0,034	0,103	-0,235	0,168	0,744
	Time 6mo	-0,083	0,073	-0,226	0,06	0,256
	Time 12mo	-0,086	0,072	-0,227	0,055	0,234
	Time 6mo x Arm	0,086	0,096	-0,102	0,273	0,369
	Time 12mo x Arm	0,08	0,093	-0,103	0,262	0,392
**Relationships[a]**	**Intercept**	**1,253**	**0,093**	**1,07**	**1,436**	**<0.01**
**(primary outcome)**	**Arm at baseline**	**0,055**	**0,121**	**-0,182**	**0,292**	**0,651**
	**Time 6mo**	**-0,069**	**0,102**	**-0,269**	**0,131**	**0,498**
	**Time 12mo**	**0,054**	**0,102**	**-0,146**	**0,254**	**0,595**
	**Time 6mo x Arm**	**-0,029**	**0,131**	**-0,286**	**0,229**	**0,828**
	**Time 12mo x Arm**	**-0,168**	**0,132**	**-0,427**	**0,091**	**0,204**
Substance abuse[a]	Intercept	0,518	0,074	0,372	0,663	<0.01
	Arm at baseline	-0,149	0,096	-0,337	0,039	0,12
	Time 6mo	-0,172	0,059	-0,288	-0,056	0,004
	Time 12mo	-0,026	0,059	-0,141	0,09	0,662
	Time 6mo x Arm	0,08	0,076	-0,068	0,228	0,29
	Time 12mo x Arm	-0,007	0,076	-0,155	0,142	0,928
Psychosis[a]	Intercept	0,998	0,107	0,787	1,208	<0.01
	Arm at baseline	-0,059	0,139	-0,331	0,213	0,67
	Time 6mo	-0,055	0,086	-0,224	0,114	0,521
	Time 12mo	-0,136	0,086	-0,304	0,031	0,111
	Time 6mo x Arm	-0,065	0,112	-0,286	0,155	0,56
	Time 12mo x Arm	0,033	0,11	-0,184	0,249	0,767

Outcome	Parameter	Estimate	Standard error	95% CI lower	95% CI upper	p value
IFIP question burden[b]	Intercept	4,392	0,2	3,999	4,784	<0.01
	Arm at baseline	0,033	0,258	-0,473	0,538	0,9
	Time 6mo	0,491	0,198	0,103	0,878	0,013
	Time 12mo	0,218	0,197	-0,167	0,603	0,268
	Time 6mo x Arm	-0,029	0,256	-0,532	0,473	0,909
	Time 12mo x Arm	0,325	0,25	-0,165	0,816	0,194
1.PC[c]	Intercept	3,1	0,253	2,604	3,597	<0.01
	Arm at baseline	0,688	0,326	0,048	1,327	0,035
	Time 6mo	0,138	0,3	-0,449	0,726	0,645
	Time 12mo	0,515	0,298	-0,07	1,1	0,084
	Time 6mo x Arm	-0,581	0,391	-1,347	0,185	0,137
	Time 12mo x Arm	-0,943	0,38	-1,687	-0,199	0,013
2. PW[c]	Intercept	8,15	0,24	7,679	8,621	<0.01
	Arm at baseline	-0,407	0,31	-1,015	0,2	0,189
	Time 6mo	0,164	0,237	-0,301	0,628	0,49
	Time 12mo	-0,221	0,235	-0,681	0,24	0,347
	Time 6mo x Arm	-0,427	0,308	-1,03	0,176	0,165
	Time 12mo x Arm	-0,396	0,303	-0,99	0,198	0,191
3. PC[c]	Intercept	3,206	0,255	2,707	3,706	<0.01
	Arm at baseline	0,127	0,329	-0,518	0,772	0,699
	Time 6mo	-0,142	0,309	-0,748	0,465	0,647
	Time 12mo	-0,076	0,301	-0,667	0,514	0,8
	Time 6mo x Arm	-0,083	0,4	-0,866	0,701	0,836
	Time 12mo x Arm	-0,097	0,389	-0,86	0,666	0,803
4. PW[c]	Intercept	8,286	0,236	7,824	8,748	<0.01
	Arm at baseline	-0,21	0,304	-0,807	0,386	0,489
	Time 6mo	-0,018	0,287	-0,58	0,544	0,95
	Time 12mo	-0,161	0,284	-0,717	0,396	0,571
	Time 6mo x Arm	-0,254	0,377	-0,992	0,484	0,5
	Time 12mo x Arm	0,043	0,368	-0,678	0,763	0,907
5. PC[c]	Intercept	4,61	0,289	4,043	5,177	<0.01
	Arm at baseline	0,314	0,371	-0,414	1,042	0,397
	Time 6mo	-0,361	0,337	-1,021	0,3	0,285
	Time 12mo	0,017	0,329	-0,629	0,663	0,959
	Time 6mo x Arm	0,469	0,43	-0,374	1,313	0,276
	Time 12mo x Arm	-0,164	0,424	-0,995	0,666	0,699
ReQoL sumscore[d]	Intercept	24,247	0,869	22,544	25,95	<0.01
	Arm at baseline	0,094	1,124	-2,11	2,297	0,934
	Time 6mo	1,679	0,812	0,087	3,27	0,039
	Time 12mo	1,535	0,834	-0,101	3,171	0,066
	Time 6mo x Arm	-1,062	1,051	-3,123	0,998	0,312
	Time 12mo x Arm	0,48	1,062	-1,601	2,562	0,651
ReQoL physical health[d]	Intercept	2,991	0,1	2,794	3,188	<0.01
	Arm at baseline	-0,006	0,129	-0,259	0,247	0,961
	Time 6mo	0,088	0,105	-0,117	0,293	0,4
	Time 12mo	-0,01	0,104	-0,214	0,193	0,921
	Time 6mo x Arm	0,063	0,133	-0,198	0,325	0,634
	Time 12mo x Arm	0,22	0,132	-0,039	0,48	0,096

Outcome	Parameter	Estimate	Standard error	95% CI lower	95% CI upper	p value
MANSA[e]	Intercept	4,543	0,153	4,243	4,842	<0.01
	Arm at baseline	-0,058	0,197	-0,444	0,328	0,768
	Time 6mo	0,02	0,171	-0,314	0,355	0,906
	Time 12mo	0,042	0,168	-0,287	0,371	0,802
	Time 6mo x Arm	0,092	0,222	-0,343	0,526	0,679
	Time 12mo x Arm	-0,024	0,216	-0,447	0,399	0,913
HoNOS sum score[f]	Intercept	8,914	0,616	7,706	10,121	<0.01
	Arm at baseline	0,167	0,806	-1,412	1,746	0,836
	Time 12 mo	0,684	0,521	-0,338	1,706	0,189
	Time 12mo x Arm	-1,733	0,671	-3,05	-0,416	0,01

Model fit statistics

Outcome	Centre level variance	Patient level variance	Residual variance	ICC	Conditional Rsq	Marginal Rsq
BASIS-24 mean score[a]	0,03	0,273	0,17	0,64	0,643	0,006
Depression functioning[a]	0,038	0,436	0,346	0,578	0,581	0,007
Emotional lability[a]	0,029	0,397	0,337	0,558	0,566	0,019
Self harm[a]	0,005	0,326	0,225	0,596	0,597	0,001
**Relationships[a]**	**0,034**	**0,312**	**0,434**	**0,443**	**0,445**	**0,004**
Substance abuse[a]	0,026	0,324	0,143	0,709	0,713	0,013
Psychosis[a]	0,016	0,689	0,312	0,693	0,694	0,004
IFIP question burden[b]	0,083	1,833	1,586	0,547	0,554	0,015
1.PC[c]	<0.001	1,907	3,697	0,34	0,347	0,01
2.PW[c]	0,265	2,471	2,333	0,54	0,556	0,036
3.PC[c]	<0.001	1,852	3,848	0,325	0,326	0,002
4. PW[c]	0,085	1,376	3,425	0,299	0,304	0,007
5.PC[c]	0,257	2,424	4,509	0,373	0,379	0,009
ReQoL sumscore[d]	2,355	37,326	27,817	0,588	0,592	0,01
ReQoL physical health[d]	0,007	0,42	0,441	0,491	0,496	0,009
MANSA[e]	<0.001	0,884	1,145	0,436	0,437	0,001
HoNOS sum score[f]	0,97	24,136	11,192	0,692	0,694	0,009

Primary outcome: bold numbers. Abbreviations: a The Behavior and Symptom Identification scale (Basis-24); b IFIP trial question: Experienced burden of mental health problems; c Perceived criticism (PC) and perceived warmth (PW) from relative: 1) How critical is he/she of you, 2) How warm is he/she towards you, 3) How disapproving is he/she of what you do?, 4) How caring is he/she of you?, 5) When he/she criticizes you. how upset do you get?; d The Recovering Quality of Life questionnaire (ReQoL-10); e The Manchester Short Assessment of Quality of Life (MANSA). One question: How satisfied are you with your life as a whole today?; f The Health of the Nation Outcome Scale (HoNOS)
